# Modulation of excitatory and inhibitory systems in autism spectrum disorder: the role of cannabinoids

**DOI:** 10.1192/j.eurpsy.2023.662

**Published:** 2023-07-19

**Authors:** S. Marini, L. D’Agostino, C. Ciamarra, A. Gentile

**Affiliations:** Mental Health, National Health Service, Termoli, Italy

## Abstract

**Introduction:**

Autism Spectrum Disorder (ASD) includes a group of developmental disabilities characterized by patterns of delay and deviance in the development of social, communicative, cognitive skills and the presence of repetitive and stereotyped behaviors as well as restricted interests (APA, 2013 DSM 5th ed.). Although the etiopathogenesis of autism has not yet been elucidated, past literature has highlighted an imbalance between glutamatergic and gamma-aminobutyric acid (GABA)-ergic neurotransmission (Harada et al. J Autism Dev Disord 2011;41:447-54.). A cortical deficiency of GABA in young people with ASD has been reported (Rojas et al. Neuroimage 2013;86:28-34.). Endocannabinoids act in numerous synapses of the central nervous system, maintaining an adequate synaptic homeostasis, preventing excess stimulation at the level of excitatory or inhibitory synapses. They therefore appear to be fundamental for the short- and long-term control of synaptic plasticity (Castillo et al. Neuron 2012;76,70-81). The endocannabinoid system appears to play an important role in some clinical presentations of autism, such as socialization. Indeed, Autism Spectrum Disorder seems to be characterized by a hypo-functionality of the endocannabinoid system (Aran et al. Mol Autism 2019;10, 2).

**Objectives:**

The present work aims to describe the current state of the art regarding the possible role of cannabinoids in the modulation of the excitatory and inhibitory systems in individuals with ASD.

**Methods:**

We carried out a search on PubMed concerning the randomized clinical trials on the modulating effect of excitatory and inhibitory cannabinoid systems in autism. Three eligible articles were found according to the purpose of the present study.

**Results:**

The results of the three articles considered highlighted a cannabinoid (CBD)-related increase in glutamate in subcortical regions (basal ganglia) and a decrease in cortical regions (dorsomedial prefrontal cortex), both in subjects with and without ASD. CBD increased GABA transmission in the subcortical regions of neurotypical subjects, while it decreased it in the same areas of the ASD group. Furthermore, CBD modulated low-frequency activity, used as a measure of brain activity and functional connectivity in the brains of adults with ASD.

**Conclusions:**

Data from the three functional MRI studies demonstrated that CBD influences cortical and subcortical connectivity on an adult sample. This effect was notable only in the ASD group but not in the controls. However, further studies are needed to confirm the results obtained so far.

**Disclosure of Interest:**

None Declared

